# How to Teach Heat Transfer More Systematically by Involving Entropy

**DOI:** 10.3390/e20100791

**Published:** 2018-10-15

**Authors:** Heinz Herwig

**Affiliations:** Institute of Thermodynamics, Hamburg University of Technology, D-21073 Hamburg, Germany; h.herwig@tuhh.de

**Keywords:** entropy, entropic potential, energy devaluation number

## Abstract

In order to teach heat transfer systematically and with a clear physical background, it is recommended that entropy should not be ignored as a fundamental quantity. Heat transfer processes are characterized by introducing the so-called “entropic potential” of the transferred energy, and an assessment number is based on this new quantity.

## 1. Introduction

This paper is not primarily about new research items, but it rather tries to improve the way in which we can pass on our knowledge about heat transfer to those young scientists who will deal with new research items in the future. 

“To improve” here means that certain aspects of heat transfer teaching can be added, while others may be modified to what is standard in today’s teaching of heat transfer.

What will be presented in the paper relates to the almost complete ignorance of how entropy can contribute to the understanding of heat transfer. There are good arguments for dealing with entropy and entropy generation when the physics of heat transfer is being explained and heat transfer situations are being assessed, as illustrated in Reference [1].

To get to the point: Heat transfer without entropy is like walking without shoes; you can do it, but something important is missing. 

## 2. Heat Transfer and Entropy 

For those who are not really familiar with entropy and its physical meaning, there are a vast number of textbooks and monographs about thermodynamics that include major parts on entropy. Special books about entropy range from easy-to-read introductions [2,3,4] to more comprehensive books [5] and very challenging studies on the topic [6,7].

**The Typical Student’s Question: What is Entropy?** Students—and perhaps engineers to an even greater extent—are used to asking direct and simple questions such as, “What is entropy?” Having asked this question, they expect a simple answer like, “Entropy is …”. Now, if the missing “…” was an already known word or phrase, entropy would be just another name for something already well known and understood. However, the “…” is much more: It is a concept with various aspects and new implications (at least for someone not familiar with entropy). Therefore, in order to understand entropy, one has to learn all about the concept behind it. This is a learning process, similar to the process by which people finally have an idea of what energy is. However, the “energy learning process” usually starts in early years of a life, while the “entropy learning process” comes late (if at all). 

**Why is Entropy Good for Heat Transfer Problems?** It is the aim of this paper to convince engineers involved in heat transfer problems that entropy and its generation rate may be of relevance in their problems and may help to better understand the physics behind them. 

With entropy, it is often not the quantity itself that is important in engineering applications but its change. As entropy generally cannot be destroyed, what may occur is either its change by transport or a generation of entropy. 

There are two aspects that can be identified to be of major significance in engineering heat transfer problems:

- entropy generation and the determination of losses
- entropy generation and the assessment of complex heat transfer processes

Here, the generation of entropy within a heat transfer process will be the important aspect with respect to entropy in heat transfer applications.

## 3. The Nusselt Number and Entropy Considerations

The conventional way to assess a heat transfer process is by applying the Nusselt number:

(1) $$\mathrm{Nu}=\frac{{\dot{q}}_{w}L}{k\Delta T}$$

This is a nondimensional number without direct reference to entropy.

From a thermodynamic point of view, heat transfer is a process by which entropy is involved in two ways (all temperatures in Kelvin):

(1) Transport of entropy over the system’s boundary at a temperature, $T_{s}$, with a rate:
  (2) $${\dot{S}}_{Q}=\frac{{\dot{Q}}_{w}}{T_{s}}=\frac{{\dot{q}}_{w}A}{T_{s}}$$
(2) Generation of entropy in the temperature field next to the system’s boundary due to the temperature difference $\Delta T=T_{s}-T_{\infty }$ with a rate:
  (3) $${\dot{S}}_{irr}={\dot{Q}}_{w}\left(\frac{1}{T_{\infty }}-\frac{1}{T_{s}}\right)={\dot{q}}_{w}A\frac{\Delta T}{T_{\infty }T_{S}}$$

The transport of entropy ${\dot{S}}_{Q}$ at the system’s boundary is a reversible process. The losses in the overall heat transfer process are characterized by the entropy generation, ${\dot{S}}_{irr}$. Thus, for ΔT=0 according to Equation (3) ${\dot{S}}_{irr}=0$, the overall heat transfer process is reversible. For ΔT=0 according to Equation (1), Nu=∞ holds, and thus a finite Nusselt number means that the heat transfer is irreversible, i.e., subject to losses.

These considerations show that heat transfer has two aspects:

- Quantity: The amount of energy transferred in the form of heat—characterized by ${\dot{q}}_{w}$
- Quality: The amount of entropy produced, i.e., how irreversible the transfer process is—characterized by ΔT

Both aspects are “combined” in the often-used heat transfer coefficient:

(4) $$h=\frac{{\dot{q}}_{w}}{\Delta T}$$

and, as Equation (1) shows, also in the Nusselt number:

(5) $$\mathrm{Nu}=h\frac{L}{k}=\frac{{\dot{q}}_{w}L}{k\Delta T}$$

This “combination” however, is hard to interpret in terms of its physical meaning. Prior to further statements about Nu, losses in a heat transfer process should therefore be analyzed and specified.

It is sometimes (cf. Reference [8]) argued that Nu is for heat transfer what the friction coefficient f is for the flow (being a measure of losses). A closer look, however, on this “analogy” shows that in both cases, there are two aspects of quantity and quality. Table 1 shows the characteristic variables with respect to both categories, with a turbulent pipe flow as an example. Cast into a nondimensional form, they result in two independent numbers that are the friction factor $f=\left(-dp/dx\right)2D/\rho u_{m}^{2}$ and the Reynolds number Re=ρumD/η for the turbulent flow. Again, the problem with the Nusselt numbers arises when Nu is assumed to be the only nondimensional number characterizing the heat transfer.

One nondimensional number is sufficient in only very special cases where both variables—$u_{m}$ and dp/dx as well as ${\dot{q}}_{w}$ and ΔT—are proportional to each other. This is the case, for example, for a fully developed laminar pipe flow with constant heat flux ${\dot{q}}_{w}$ and for the flow fRe=Po=64 and Nu=4.36. From this “analogy”, one can loosely conclude that the Nusselt number Nu corresponds to the Poiseuille number Po. However, this is limited to special cases when quantity ($u_{m}$, ${\dot{q}}_{w}$) and quality (dp/dx, ΔT) are completely linked.

For a general discussion of the problems associated with the Nusselt number and its definition, see Reference [9].

## 4. Losses in a Heat Transfer Process

Energy transferred in the form of heat has a certain quality, and it can be lost partly or completely by the transfer process. The quality of the transferred energy can best be quantified by the “exergy” concept in which energy is divided into two parts: the exergy and the anergy (see, for example, Reference [10]). Exergy, also called “available work”, is the maximum theoretical work obtainable from the energy interacting with the environment to equilibrium and is thus the precious part of the energy.

Generally, energy E is split into two parts:

- $E^{e}$: exergy as the part that can be completely transferred to the internal energy of the ambient by work (also called “available work”)
- $E^{a}$: anergy as all of E that is not exergy.

According to this concept, losses in an irreversible heat transfer are losses of exergy, i.e., its conversion into anergy. When this conversion rate is ${\dot{E}}_{C}$ with $\dot{E}$ as the energy flux $\dot{E}={\dot{Q}}_{w}$ in the heat transfer process, the rate by which exergy gets lost (is converted) according to the Gouy–Stodola theorem is as follows:

(6) $${\dot{E}}_{C}=T_{0}{\dot{S}}_{irr}=\frac{{\dot{q}}_{w}AT_{0}\Delta T}{T_{\infty }T_{s}}$$

where $T_{0}$ is the temperature of the environment and ${\dot{S}}_{irr}$ is the entropy generation rate according to Equation (3).

It is important to note that losses in a heat transfer process depend on the temperature level $T_{\infty }$ on which the process occurs—the higher the temperature, the lower the losses when transferring energy by a certain rate ${\dot{Q}}_{w}={\dot{q}}_{w}A$. This alone shows that the Nusselt number cannot be a measure of heat transfer losses as $T_{\infty }$ does not occur in it, cf. Equation (5). The following example may further illustrate this point.

**Example 1: Heat Transfer in Power Cycles**

Heat transfer is an intrinsic part of a power cycle and happens at the highest and the lowest temperatures of the cycle. In this example, the quality of heat transfer in a conduit component on the high temperature level of the cycle will be compared for a conventional steam power cycle (SPC) and an organic Rankine cycle (ORC). In these cycles, there is a heat transfer into the working fluid within a range of high temperatures. Although an overall efficiency of the power cycle would incorporate the thermodynamic mean temperature, we will compare the performance of the single conduit component when it is part of the overall heat transfer unit at the “high temperature end”.

In a steam power cycle, heat transfer to the water vapor typically occurs at a maximum temperature $T_{s}=$ 600 °C = 873.15 K. In an ORC with ${\mathrm{NH}}_{3}$ as the working fluid, the typical corresponding temperature is $T_{s}=100$ °C = 373.15 K.

Assuming the same Nusselt number Nu=100 (turbulent pipe flow) and the same ${\dot{q}}_{w}=10^{3}\;W/m^{2}$ and L=0.1 m for both cases, the temperature difference for ${\mathrm{NH}}_{3}$ is larger by a factor of $k_{H_{2}O}/k_{{\mathrm{NH}}_{3}}$ than that for water. As $k_{H_{2}O}/k_{{\mathrm{NH}}_{3}}\approx 2$, we have ΔT=11 K for water and ΔT=22 K for ${\mathrm{NH}}_{3}$. With these numbers, and assuming $T_{0}=300\;K$, we get the results in Table 2.

With the same Nusselt number Nu=100 for both cases, which might suggest an equally “good” heat transfer, the devaluation of the transferred energy is very different. For the high temperature SPC, the energy is devaluated by 0.44%, and 0.67% of the exergy gets lost, whereas for the low temperature ORC, the devaluation is 5%, and 24% of the exergy gets lost. The 0.67% and 24% less exergy after the heat transfer means that the amount of available work is lost for a conversion into mechanical energy at the turbine of the cycles.

## 5. The Entropic Potential of Energy

**Figure 1:**
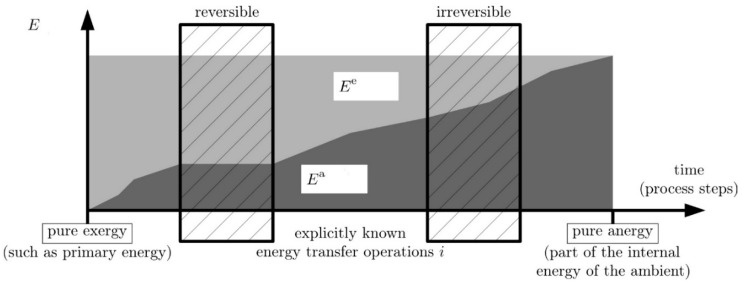
Energy devaluation by consecutive energy transfer operations illustrated by the decrease of exergy during the energy transfer operations. Progress in time for finite energies (progress in process steps for finite energy rates). Figure adopted from [11].

The energy here is analyzed while transferred in the form of heat across a system boundary. Once, i.e., some process steps prior to the one observed here, it started as primary energy. As such, it was pure exergy but finally, i.e., after some more process steps, it becomes part of the internal energy of the ambient and thus turns into pure anergy. Figure 1 shows this devaluation of the energy from its state as primary energy to being part of the ambient internal energy.

The initial energy in progressive times can either be left as it is or it can undergo consecutive energy transfer operations. During such a transfer operation, it will be partly and further devaluated (assuming transfer operations have already occurred) when the transfer operation is irreversible. This devaluation manifests itself in a (further) decrease of the exergy part in E. Only when a transfer operation would be reversible would $E^{e}$ not decrease further. In Figure 1, two energy transfer operations are assumed to be explicitly known (one reversible, one irreversible). Before, between, and after these two operations, the energy E undergoes further—yet not explicitly known—transfer operations. Finally, the energy E is part of the ambient internal energy and then, as per definition, becomes 100% anergy.

For a single transfer operation numbered i, which may be a heat transfer operation, there is the exergy loss, cf. Equation (6):

(7) $$E_{C,i}=T_{0}S_{irr,i}$$

where $S_{irr,i}$ is the entropy generation during the transfer operation number *i*.

For the sum of all single transfer operations that completely devaluates the energy E from being 100% exergy to 100% anergy then:

(8) $$E=E_{TC}=\underset{i}{\sum }E_{C,i}=T_{0}\underset{i}{\sum }S_{irr,i}=T_{0}S_{irr}^{TC}$$

Here, the index TC means total conversion, and $S_{irr}^{TC}$ is the entropy increase of the ambient when E becomes part of its internal energy. 

This quantity from now on will be called “entropic potential of the energy” E, defined as follows:

(9) $$S_{0}=\frac{E}{T_{0}}\;or\;{\dot{S}}_{0}=\frac{\dot{E}}{T_{0}}$$

and will be used in the subsequent considerations as the crucial reference quantity. For more information about this concept see References [11,12].

## 6. Assessment of Losses in a Heat Transfer Process

**Figure 2:**
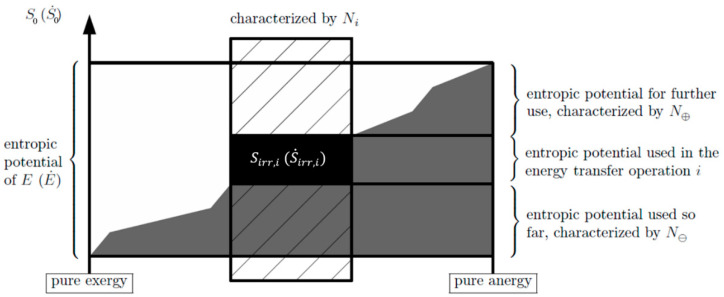
The entropic potential and its use on the way the energy becomes part of the internal energy of the ambient, i.e., contribution of an energy transfer operation *i*. Figure adopted from [11].

In Figure 1, the link to the devaluation of an energy transferred in a (heat transfer) process *i* is obvious: It is the amount of entropic potential used in it. This is further illustrated in Figure 2, where a single transfer process *i* is put into the perspective of the entire devaluation chain of the energy E from pure exergy to pure anergy.

The partial devaluation of the energy E in the transfer process *i* is quantified and thereby assessed by a nondimensional number $N_{i}$ called the “energy devaluation number”. Entropy generation occurs in the process *i* by which the energy E is transferred, which is referred to the entropic potential of E:

(10) $$N_{i}\equiv \frac{S_{irr,i}}{S_{0}}=\frac{T_{0}S_{irr,i}}{E}\left(=\frac{E_{C,i}}{E}\right)$$

When the transfer operations are not time-limited but are part of a steady process, $S_{irr,i}$ and $S_{0}$ are replaced by their rates (change per time) so that the energy devaluation number in these cases is as follows:

(11) $$N_{i}\equiv \frac{{\dot{S}}_{irr,i}}{{\dot{S}}_{0}}=\frac{T_{0}{\dot{S}}_{irr,i}}{\dot{E}}\left(=\frac{{\dot{E}}_{C,i}}{\dot{E}}\right)$$

This number is always between 0 and 1 or interpreted as the percentage of the entropic potential used in a transfer process *i* between 0% and 100%. The two limits are

- $N_{i}=0$ or $N_{i}=0\%$: reversible energy transfer operation
- $N_{i}=1$ or $N_{i}=100\%$: energy transfer operation that completely devaluates the energy (rate).

When the overall devaluation of the energy E or $\dot{E}$ prior to the energy transfer operation under consideration is given by the energy devaluation number $N_{⊖}$ and that after the energy transfer operation is given by $N_{⊕}$, the overall devaluation corresponds to:

(12) $$N_{⊖}+N_{i}+N_{⊕}=1$$

In Figure 2, the energy devaluation numbers $N_{i}$, $N_{⊖}$, and $N_{⊕}$ are introduced and show how a single transfer process *i* is part of the entire devaluation chain.

In order to determine specific values of $N_{i}$ for a certain energy transfer operation, its entropy generation (rate) must be known. There are basically two ways to determine $S_{irr,i}$ or ${\dot{S}}_{irr,i}$ for an energy transfer operation.

The first way is determining them from a global entropy balance by taking into account the inflowing and outflowing entropies as well as that transferred by energy flows in the form of heat into or out of the system. The second way to determine entropy generation is locally, using the velocity gradients in the flow field and the temperature gradients in the temperature field and then integrating them over the entire flow and temperature fields. However, this can only be done with numerical solutions of the problem. The first method is called the “indirect method” of determining entropy generation, while the second way is called the “direct method” (see Reference [13] for more details). Here “direct” means that the local entropy generation is integrated “directly” in order to determine $S_{irr,i}$.

Once $S_{irr,i}$ or ${\dot{S}}_{irr,i}$ is known, $N_{i}$ can be determined as the reference quantities are the known energy (rate) and the ambient temperature (thermodynamic temperature in Kelvin (K)).

The devaluation number $N_{i}$ resembles the often-used exergetic efficiency $\eta_{ex,i}$, defined as $\eta_{ex,i}=1-E_{C,i}/E^{e}$ so that $1-\eta_{ex,i}=E_{C,i}/E^{e}=N_{i}E/E^{e}$. The reference quantity in $\eta_{ex,i}$ is $E_{i}^{e}$, while it is E in $N_{i}$. Only with E as a reference quantity can operation i be evaluated as part of the whole devaluation chain with respect to the energy E.

## 7. Applying the Energy Devaluation Number

When it comes to optimizing a process that often comprises several single energy transfer operations, the energy devaluation number of the whole process should be minimum while maintaining its original purpose. This implicitly assumes that the least energy devaluation is the optimization target as it will often be the case in processes in which the exergy and its use is the crucial objective. Nevertheless, there may be additional constraints that have to be taken into account. 

The devaluation number concept is applicable for a wide variety of single or combined energy transfer operations, ranging from a single unit operation within one component (e.g., in an evaporator, condenser, heater, cooler, etc.) to the whole component as part of a cycle (power plant, cooling cycle, etc.).

Two examples (first shown in Reference [11]) can be given for which the least energy devaluation is the target of optimization. The first one is a whole steam power cycle, while the second is a detailed analysis of the cold side of a heat exchanger. These examples should show that the energy devaluation numbers can be applied on very different detail levels of the “energy path from exergy to anergy”. They can be as comprehensive as that for a whole boiler in the steam power cycle or as detailed as that for the cold side of a heat exchanger (which may be implemented, for example, in a steam power cycle). Whichever level is chosen, the corresponding energy devaluation number for a single component or operation is one $N_{i}$ in the overall energy devaluation chain where $\sum_{i}N_{i}=1$ holds (see Equation (12)).

**Example 2: Assessment of a Steam Power Cycle**

**Figure 3:**
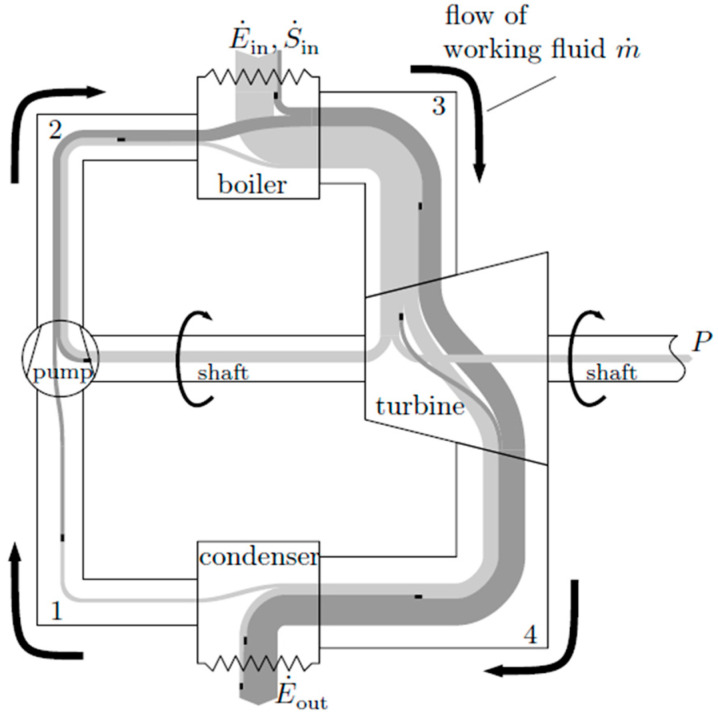
Energy transfer in a steam power cycle (qualitative). Light grey: exergy, dark grey: anergy, black box: entropy generation. Figure adopted from [11].

In Figure 3, a schematic diagram of the energy transfer operations in a steam power cycle is shown. The process as a whole is characterized and assessed by the four transfer components—pump (p), boiler (b), turbine (t), condenser (c)—and their performance. For simplicity, the exergy losses in the pipes are assigned to the components between the corresponding interfaces 1 to 4; those in the ambient are assumed to be part of the condenser exergy losses. In Reference [14], it is shown how this concept can be applied to gas turbine loss considerations.

Applying the indirect method to determine the entropy generation of each component, ${\dot{S}}_{irr,i}$ energy devaluation numbers for all four components can be determined as shown in Table 3. The corresponding assessment number for the whole cycle is as follows:

(13) $$N_{cycle}=N_{p}+N_{b}+N_{t}+N_{c}=\frac{\left({\dot{S}}_{out}-{\dot{S}}_{in}\right)T_{0}}{{\dot{E}}_{in}}$$

or rewritten as follows:

(14) $${\dot{E}}_{out}={\dot{E}}_{in}-P,\;{\dot{E}}_{out}={\dot{S}}_{out}T_{0}$$

(15) $$N_{cycle}=1-\underset{I}{\underset{︸}{\frac{P}{{\dot{E}}_{in}}}}-\underset{\mathrm{II}}{\underset{︸}{\frac{{\dot{S}}_{in}T_{0}}{{\dot{E}}_{in}}}}$$

This example shows how information about the occurrence of energy devaluation gets lost when detail levels of the analysis are changed. When all four components are analyzed individually like in Table 3, energy devaluations can be attributed to the single components. However, when they are combined, in order to end up with the $N_{cycle}$ according to Equation (13) or Equation (15), this information gets lost. Note that in Equation (15), the term I corresponds to the thermal coefficient for performance with respect to the whole cycle, and term II represents $N_{⊖}$ introduced in Equation (12). According to Equation (12), term I also represents $N_{⊕}$, which means that the exergy P will eventually be completely lost (when used for subsequent processes). The entropic potential ${\dot{E}}_{in}/T_{0}$ will then be used completely and nothing will be left for further use.

**Example 3: Assessment of the Cold Side of a Heat Exchanger**

With this example, a very detailed analysis will be given about the heat transfer performance on the cold side of a heat exchanger. Here, a mass flow rate $\dot{m}$ is heated by a constant energy transfer in the form of a constant heat flux per pipe length ${\dot{q}}_{w}^{\prime }$ such that there is a constant gradient in bulk temperature $dT_{m}/dx$. The question to be addressed is whether a rough wall might perform better than a smooth wall and to determine the degree of roughness that performs best in this case. The idea behind this question is that a better mixing of the fluid by wall roughness may decrease the exergy loss due to heat conduction such that it outweighs the increase of exergy loss due to increased frictional losses.

**Figure 4:**
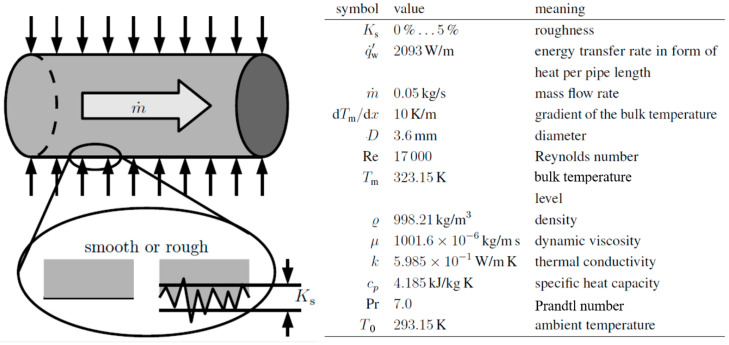
Details at the cold side of a heat exchanger that might operate with rough instead of smooth walls, assuming constant properties with values at T=293.15 K. Figure adopted from [11].

Figure 4 is a sketch of this situation, along with details with respect to the operational parameters and fluid properties.

In a conventional approach, the performance assessment might have been based on the often-used and still-popular thermohydraulic performance parameter proposed by Gee and Webb [15], which is as follows:

(16) $$\eta =\frac{\mathrm{St}/{\mathrm{St}}_{0}}{{\left(f/f_{0}\right)}^{1/3}}$$

Here, the Stanton number ${\mathrm{St}}_{0}$ and the friction factor $f_{0}$ correspond to the smooth wall case; St and f are these parameters when wall roughness $K_{s}$ is non-zero.

The influence of wall roughness for increasing roughness heights $K_{s}$ is directly accounted for in f:

(17) f−1/2=−2log10(Ks3.7+2.51Ref−1/2)

as proposed by Reference [16] and indirectly in St:

(18) St=f/8(Re−1000)Re(1+12.7(f/8)1/2(Pr2/3−1))

as proposed by Reference [17] and recommended in Reference [8] for tubes with wall roughness type of sand-grain.

**Figure 5:**
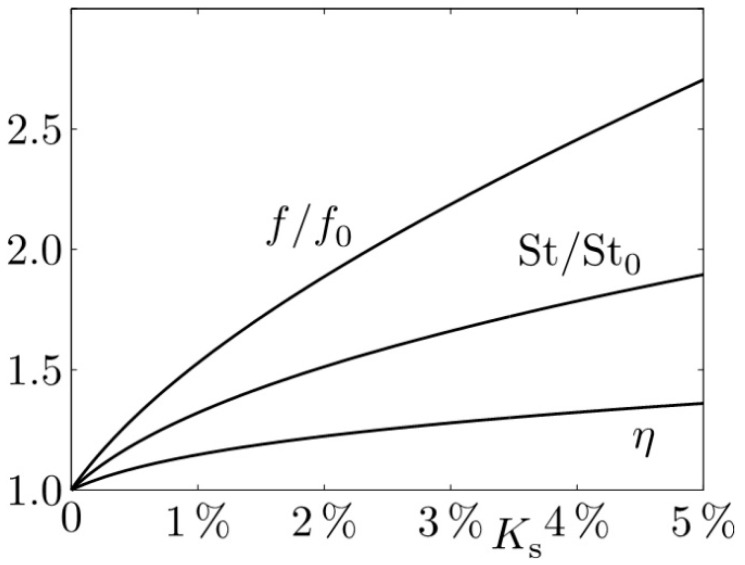
Roughness effects at the cold side of the heat exchanger, see Figure 4, in terms of f, St, and η. Figure adopted from [11].

Figure 5 shows $f/f_{0}$, $\mathrm{St}/{\mathrm{St}}_{0}$ and their combination as η according to Equation (16). With increasing $K_{s}$, there is a monotonous increase in all three quantities. In particular, η increases above η=1, which is claimed to be an improvement, although without physical explanation. According to these results, $K_{s}$ should be as high as possible.

Alternatively, this problem can be assessed by applying the energy devaluation number $N_{i}$, which will then account for the exergy loss, i.e., entropy generation due to the dissipation of mechanical energy in the flow field, that due to heat conduction along finite temperature gradients in the temperature field, and the entropic potential of the transferred energy.

Here, entropy is not only generated in the temperature field but also by the flow, dissipating mechanical into internal energy. 

In a direct method approach, CFD (computational fluid dynamics) results with respect to the flow and temperature fields could provide the local rates of entropy generation per length (see Reference [18] for all the details). Here, however, we relate these generation rates to the friction factor f and Stanton number St in a way proposed in Reference [19], p. 80, which is as follows:

(19) $${\dot{S}}_{irr,D}^{\prime }=\frac{32{\dot{m}}^{3}}{\pi^{2}\rho^{2}T_{m}D^{5}}f$$

(20) $${\dot{S}}_{irr,C}^{\prime }=\frac{{\dot{q}}_{w}^{\prime 2}}{\pi kT_{m}^{2}\mathrm{RePr}}{\mathrm{St}}^{-1}$$

Equations (19) and (20) show that the entropy generation rate per length due to dissipation increases when *f* increases, but the entropy generation due to heat conduction decreases when St is increased, and the heat transfer is thus improved.

**Figure 6:**
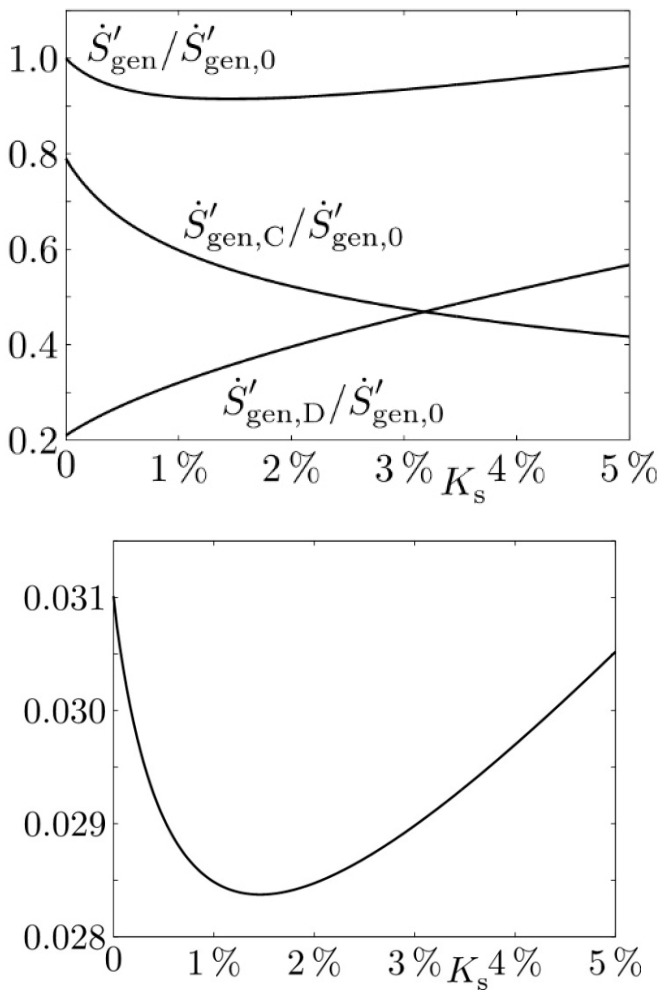
Roughness effects at the cold side of the heat exchanger, see Figure 4, in terms ${\dot{S}}_{irr,D}^{\prime },$ ${\dot{S}}_{irr,C}^{\prime }$, and $N_{i}$. Top: Entropy generation, bottom: Energy devaluation number. Figure adopted from [11].

Figure 6(top) shows the influence of wall roughness on the single contributions ${\dot{S}}_{irr,D}^{\prime }$ and ${\dot{S}}_{irr,C}^{\prime }$ and on their sum ${\dot{S}}_{irr}^{\prime }$. For a smooth wall, the entropy generation due to dissipation is appreciably smaller than that due to heat transfer. However, this is no longer true for increasing wall roughness. In accordance with the physics of the problem ${\dot{S}}_{irr,D}^{\prime }$ increases while ${\dot{S}}_{irr,C}^{\prime }$ is reduced. Because these effects are antipodal, the sum may have an extremum, which is a minimum at $K_{s}=1.5\%$, as can be seen in Figure 6(bottom). Very different from the results given by η, a distinct optimum appears here as a result of two counteracting effects with a clear physical interpretation in terms of lost exergy.

Moreover, $N_{i}$ has a rational background; $N_{i}\approx 0.03$ means that about 3% of the entropic potential of the transferred energy are used by this heat transfer operation i on the cold side of the heat exchanger.

## 8. Conclusions

Teaching heat transfer as systemically as possible and with a clear physical background not only helps prepare students for a successful career in industry, but it can also set the starting point for heat transfer research on an advanced level—the “walking with shoes” as mentioned earlier—by seriously combining heat transfer and entropy considerations with all their implications and not just with respect to the assessment of heat transfer processes.

This paper may at least prompt open-minded teachers of heat transfer to seriously consider including entropy in the teaching of this technically important subject. 

## Figures and Tables

**Table 1:** Characteristic quantities for fully developed turbulent pipe flow with heat transfer. $u_{m}$: mean velocity, dp/dx: pressure gradient.

	Pipe Flow	Additional Heat Transfer
quantity	$u_{m}$	${\dot{q}}_{w}$
quality	dp/dx	ΔT

**Table 2:** Energy devaluation (exergy losses) for a conduit component in power cycles.

Fluid (Cycle)	Nu	${\dot{E}}_{c}/\dot{E}$	${\dot{E}}_{c}/{\dot{E}}^{e}$
Water (SPC)	100	0.0044	0.0067
HN3 (ORC)	100	0.05	0.24

**Table 3:** Energy devaluation numbers $N_{i}$ for the four energy transfer components of the steam power cycle sketched in Figure 3.

$N_{i}$	Energy Transfer		$N_{i}$
$N_{p}$	adding mechanical energy in a pump	$\dot{m}\left(s_{2}-s_{1}\right)$	$\dot{m}\left(s_{2}-s_{1}\right)T/{\dot{E}}_{in}$
$N_{b}$	adding thermal energy in a boiler	$\dot{m}\left(s_{3}-s_{2}\right)-{\dot{S}}_{in}$	$\left(\dot{m}\left(s_{3}-s_{2}\right)-{\dot{S}}_{in}\right)T/{\dot{E}}_{in}$
$N_{t}$	extracting mechanical energy in a turbine	$\dot{m}\left(s_{4}-s_{3}\right)$	$\dot{m}\left(s_{4}-s_{3}\right)T/{\dot{E}}_{in}$
$N_{c}$	extracting thermal energy in a condenser	$\dot{m}\left(s_{1}-s_{4}\right)+{\dot{S}}_{out}$	$\left(\dot{m}\left(s_{1}-s_{4}\right)+{\dot{S}}_{out}\right)T/{\dot{E}}_{in}$

## Nomenclature

A	heat transfer area (m^2^)
D	pipe diameter (m)
$\dot{E}$	energy flux (W)
${\dot{E}}_{C}$	energy conversion rate (W)
${\dot{E}}^{e}$	exergy flux (W)
f	friction factor (-)
h	heat transfer coefficient (W/m^2^ K)
k	thermal conductivity (W/mK)
L	characteristic length (m)
$\dot{m}$	mass flux (kg/s)
$N_{i}$	energy devaluation number (-)
Nu	Nusselt number (-)
p	pressure (Pa)
${\dot{q}}_{w}$	wall heat flux (W/m^2^)
${\dot{q}}_{w}^{\prime }$	wall heat flux per unit length (W/m^3^)
$Q_{w}$	energy transferred in form of heat (J)
${\dot{Q}}_{w}$	heat transfer rate
Re	Reynolds number (-)
S	entropy (J/K)
$\dot{S}$	entropy transfer rate (W/K)
$S_{0}$	entropic potential (J/K)
St	Stanton number (-)
T	local temperature (K)
$T_{0}$	temperature of the environment (K)
$T_{s}$	temperature at the system’s boundary (K)
$T_{\infty }$	temperature away from the system’s boundary (K)
ΔT	temperature difference (K)
μ	dynamic viscosity (kg/ms)
ρ	density (kg/m^3^)
